# Subcellular localization of anthracyclines in cultured rat cardiomyoblasts as possible predictors of cardiotoxicity

**DOI:** 10.1007/s10637-015-0276-9

**Published:** 2015-08-14

**Authors:** Kazimierz Studzian, Krzysztof Kik, Malgorzata Lukawska, Irena Oszczapowicz, Malgorzata Strek, Leszek Szmigiero

**Affiliations:** Department of Nucleic Acids Biochemistry, Medical University of Lodz, 251 Pomorska St., 92-213 Lodz, Poland; Institute of Biotechnology and Antibiotics, 5 Staroscinska St., 02-516 Warsaw, Poland

**Keywords:** Formamidinoanthracyclines, Cellular uptake, Subcellular localization, Rat cardiomyoblasts

## Abstract

In this study, we compared the cellular uptake, intracellular localization and cytotoxicity of two groups of anthracycline derivatives in cultured H9c2(2-1) rat cardiomyoblasts. The first group consisted of doxorubicin (DOX) and two of its derivatives containing a formamidino group (–N = CH–N<) at the C-3′ position with a morpholine (DOXM) or a hexamethyleneimine (DOXH) ring. The second group consisted of daunorubicin (DRB) and its derivatives containing a morpholine (DRBM) or a hexamethyleneimine (DRBH) ring. DOXH and DRBH were taken up by cardiomyoblasts more efficiently than estimated for other tested anthracyclines. The cellular uptakes of DOXM and DRBM were reduced compared to those of the parent compounds. Applied structural modifications of DOX and DRB influenced the subcellular localization of the tested derivatives. DOX and DOXH were localized primarily in nuclei, whereas the other anthracyclines were found in the nuclei and cytoplasm. The percentages of the compounds that accumulated in the nuclei were 80.2 and 54.2 % for DOX and DOXH, respectively. The lowest nuclear accumulation values were observed for DRBM (19.9 %), DRBH (21.9 %) and DOXM (23.7 %). The ability of anthracyclines to accumulate in the nuclei correlated with their DNA binding constants (r = 0.858, *P* = 0.029). A correlation was found between the accumulation of the tested anthracyclines in the nuclei of cardiomyoblasts and their cardiotoxicity in vivo, which was observed in our previous study. We suggest that cytotoxicity and the anthracycline accumulation level in the nuclei of cultured cardiomyoblasts could be used for early prediction of their cardiotoxicity.

## Introduction

Anthracyclines have received significant attention due to their effectiveness and extensive use as anticancer agents. At present, the clinical use of these drugs is offset by drug resistance in tumors and cardiotoxicity [1, 2]. One of the many possible strategies for improving the therapeutic effectiveness of anthracyclines is the synthesis of new analogs with modified structures. Structural modifications of the anthracycline molecule may change many biological properties of these drugs, such as cellular uptake, subcellular localization, and cellular target affinity, which may affect the drugs’ selectivity toward cancer cells [3]. Modifications at the C-3′ position of the daunosamine moiety of anthracycline may result in improvement of some biological properties that are important for the anticancer action of these compounds [4–7].

Formamidinoanthracyclines are a new group of chemically modified anthracyclines. The most important feature of this novel class of anthracyclines is their lower cardiotoxicity compared to that of the parent compounds; this lower cardiotoxicity has been demonstrated in animal models [4, 8]. Although the low cardiotoxicity of these compounds seems to be a very promising feature, how modification of the daunosamine group of anthracycline at the C-3′ position produces this effect remains unclear. One possible method to reduce the cardiotoxicity of anthracyclines may be to reduce their accumulation in cardiac cells.

Heart tissue was demonstrated to have a small subpopulation of immature myocytes produced by stem-like cells [9, 10]. Such cycling cardiogenic precursors may be more sensitive to antiproliferative drugs than terminally differentiated cardiomyocytes, and their drug-induced death may play an important role in the cardiotoxic effects of anthracyclines [11]. Therefore, cardiac myoblasts in the form of rat embryonic H9c2(2-1) cells may be a good model for assessing the cardiotoxic properties of potential anticancer agents.

**Fig. 1:**
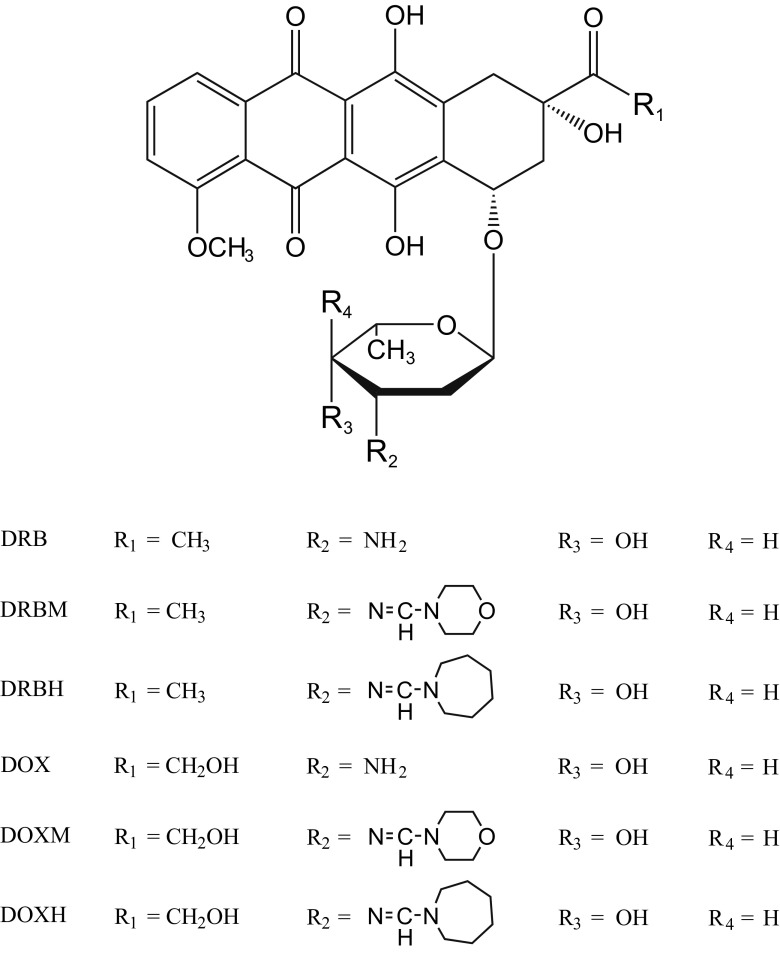
Structures of the tested anthracyclines

In this study, we attempted to determine whether the cytotoxicity of some doxorubicin (DOX) and daunorubicin (DRB) analogs, which were modified in the daunosamine moiety at the C-3′ position by the introduction of a formamidino group containing a morpholine or hexamethyleneimine ring (see Fig. 1 for structures), in cultured rat cardiomyoblasts is related to their cellular uptake and cellular localization.

## Materials and methods

### Drugs

DOX, DRB and their derivatives containing a formamidino group with a morpholine ring (DOXM and DRBM) or a hexamethyleneimine ring (DOXH and DRBH) were synthesized at the Institute of Biotechnology and Antibiotics in Warsaw [12]. Anthracyclines and their derivatives were dissolved in water and used immediately for each experiment.

### Cell lines and cell culture

Rat DB1X cardiomyoblasts (H9c2(2-1) were purchased from the European Collection of Cell Cultures (ECACC). The cells were cultured in Dulbecco’s modified Eagle’s medium supplemented with 10 % heat-inactivated fetal calf serum and 2 mM glutamine (all from Sigma-Aldrich) in a humidified 5 % CO_2_ atm at 37 °C. The cells were maintained as recommended by the supplier at a low density to avoid confluency and myoblast loss. All experiments were performed using cells at passages 4–27.

### Cytotoxicity assay

The cytotoxic activity of the drugs was assayed by measuring their inhibitory effects on H9c2(2-1) cell proliferation. To measure the cytotoxicity of doxorubicin, daunorubicin, and their analogs, the tetrazolium dye method was used [13]. Cells were seeded at 1 × 10^4^ cells/well in 48-well plates. After 24 h, the cells were incubated with different concentrations of anthracyclines for 72 h in triplicate in a final volume of 1 ml. Then, 50 μl of a sterile aqueous solution of 3-[4,5-dimethylthiazol-2-yl]-2,5-diphenyl-tetrazolium bromide (5 mg/ml) (Sigma-Aldrich) was added to each well for an additional 3 h. The blue formazan precipitate was dissolved in DMSO (Sigma-Aldrich), and the absorbance of solutions was measured at 540 nm. For each experiment, determinations were performed in three replicates. The IC_50_ values (the anthracycline concentration effective at inhibiting cell growth after exposure to the drug for 72 h) were read from survival curves, which were fitted to an exponential equation using Xact v. 7.2, a statistical software tool by SciLab (Germany).

### Cell cycle analysis

The cell cycle was analyzed by flow cytometry using a Becton-Dickinson FACSCanto II flow cytometer. H9c2(2-1) cells were seeded at 1 × 10^4^ cells/cm^2^ in 10 cm^2^ culture flasks. After the cells were allowed to grow for 24 h, the medium was replaced and the cells were treated with an equitoxic anthracycline concentration corresponding to IC_50_. Treatment was performed for 24 h, and then the cells were washed in PBS and trypsinized. After the cells were washed in PBS again, they were fixed in 70 % ethanol and incubated for 1 h on ice. Fixed cells were washed twice in PBS, resuspended in commercially available propidium iodide staining solution and then subjected to flow cytometric assay. The percentages of H9c2(2-1) cells present in G1, S, and G2 phases of the cell cycle after drug treatment were analyzed using FACSDiva 6.1 software.

### Cellular uptake

Anthracycline uptake was estimated by fluorimetric measurements after the drugs were extracted with an ethanol/HCl mixture as described by Riganti et al. [14]. H9c2(2-1) cells (2 × 10^5^) were seeded onto 40 × 11 mm tissue culture dishes for 24 h. Then, the cells were treated in growth medium with 10 μM anthracycline solution at 37 °C for 1 h. After this time, the cells were gently scraped and suspended in 5 ml of ice-cold PBS. The cell suspension aliquots were centrifuged at 1500 rpm for 5 min and then washed twice in ice-cold PBS. Next, cell pellets were collected and resuspended in 2 ml of a 1:1 mixture of ethanol:0.3 M HCl, and the fluorescence intensities of these suspensions were measured at 20 °C using a Perkin-Elmer LS 55 spectrofluorimeter. The optimal excitation and emission wavelengths were 485 and 590 nm, respectively. To estimate the drug concentrations, calibration curves in the concentration range of 0.01–0.5 μM were prepared. Because the fluorescence was quenched slightly in the presence of cells, each point of the calibration curves was measured in the presence of 2 × 10^5^ cells suspended in a mixture of ethanol/HCl.

### Subcellular localization of anthracyclines

The subcellular localization of DOX, DRB and their analogs was examined using an Olympus BX-43 fluorescence microscope. H9c2(2-1) cells (2 × 10^4^) were seeded onto 40 × 11 mm tissue culture dishes for 24 h. Then, the cells were incubated for 1 h at 37 °C in 2 ml of fresh growth medium supplemented with 10 μM drug. After incubation, the cells were washed in 4 ml of cold PBS, covered with a 24-mm cover glass, and observed at 400-fold magnification in visible light (dark field) and after excitation with a wide green filter. Cell images were acquired with an Olympus DP50 camera using the same exposure parameters for each image and saved in TIFF format as RGB images. Cell images were then processed by graphic software (Micrografx Picture Publisher v. 10) to prepare images suitable for densitometric measurements. First, the red channel for each image was separated and inverted to obtain images where areas corresponding to high fluorescence intensity became dark. Then, the part of the cell image corresponding to the nucleus was cut off and transferred above the part corresponding to the cytoplasm. This method allowed an image consisting of the separated nucleus and cytoplasm to be obtained. Such an image was then used as a path for densitometry. To estimate the percentages of drugs localized in the cytoplasm and in the nucleus, densitometric analysis was performed using GelScan v. 1.45 software (Kucharczyk T.E.).

### Affinity of anthracyclines to DNA

The binding of the anthracyclines to double-stranded calf thymus DNA was tested by the ethidium bromide displacement assay [15]. This method is based on the competition between the tested compound and ethidium bromide for DNA intercalation sites. The fluorescence intensity of ethidium bromide increases upon DNA binding. Addition of a compound displacing intercalated ethidium bromide leads to quenching of the fluorescence of the DNA/ethidium bromide complex. The percentage of initial fluorescence was plotted against the concentration of the added compound, and the IC_50_ value was read. The IC_50_ value is defined as the concentration of added compound required to reduce the fluorescence of the DNA/ethidium bromide complex to 50 %. IC_50_ values were then used to calculate the apparent binding constants of the anthracyclines to DNA.

### Statistical analysis

The data were analyzed using the SigmaPlot v. 12.5 statistics package (Systat Software, Inc. Chicago, USA). All the values in this study were expressed as the mean ± SD from at least three independent experiments. If no significant differences were detected between variances as assessed by the Snedecor–Fisher test, then the differences were compared by two-tailed Student’s *t*-test. The results were considered significant when *P* < 0.05. The relation between the DNA binding constants and nuclear localization of the anthracyclines was assessed by linear regression analysis.

## Results

### Cytotoxic activities of anthracyclines

The cytotoxicity potencies of DOX, DRB, and their analogs in H9c2(2-1) rat cardiomyoblasts exposed to the drugs for 72 h are presented in Table 1. All the tested anthracyclines were effective in inhibiting H9c2(2-1) cell proliferation. The IC_50_ values measured for doxorubicin and its derivatives were in the range of 35–138 nM. The most toxic compound in this group was the parent compound DOX, which exhibited approximately 4 times higher cytotoxic activity than DOXM and 2.5 times higher than DOXH. The IC_50_ values for daunorubicin derivatives were in the range of 75–626 nM. In this series of anthracyclines, the parent daunorubicin was the most cytotoxic drug, exhibiting 8 and 2 times higher activity compared to its analogs DRBM and DRBH, respectively.

**Table 1:** Cytotoxicity in rat cardiomyocytes, cellular and nuclear uptake of anthracycline derivatives, and their DNA binding constant values

Compound	Cytotoxicity IC_50_ (nM)	*P*	Cellular uptake nmoles/10^6^ cells	% of drug localized in the nucleus	K_app_ × 10^5^ [M^-1^]	logP*
DOX	35 ± 4		890 ± 42	80.2 ± 3.1	18.1 ± 0.41	-0.644
DOXM	138 ± 18	< 0.001	690 ± 21	23.7 ± 1.0	0.90 ± 0.12	-0.995
DOXH	87 ± 7	< 0.001	1330 ± 120	54.2 ± 6.2	7.30 ± 0.14	1.459
DRB	76 ± 7		3230 ± 153	35.8 ± 4.1	11.7 ± 0.22	-1.344
DRBM	626 ± 18	< 0.001	618 ± 51	19.9 ± 1.4	0.64 ± 0.03	-0.791
DRBH	172 ± 14	< 0.001	5640 ± 168	21.9 ± 2.1	4.1 ± 0.08	0.758

Cytotoxicity was determined by MTT assay. All IC_50_ values are the means of six independent experiments ± S.D. *P* values refer to the significances of differences between parent compounds and their analogs as analyzed by Student’s *t*-test. The data for cellular uptake are the means of 7 independent experiments ± S.D. The data presenting the % of drug in the nucleus are the means of 25 estimations for individual cells ± S.D. The DNA binding constant values are presented as the means of three independent estimations ± S.D. */logP is the partition coefficient calculated by Chem3D Ultra v. 6 software (Cambridge Soft).

### Cell cycle analysis

**Fig. 2:**
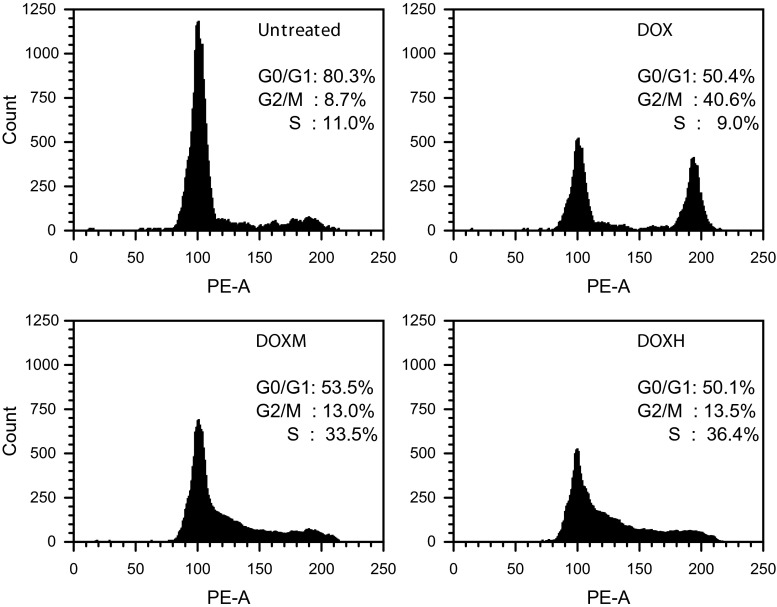
H9c2(2-1) cardiomyoblasts arrest in G2/M and S phases after anthracycline treatment. Cells were treated for 24 h with equitoxic concentrations of each drug that inhibited 50 % proliferation of cells and were subjected to flow cytometric analysis

Cell cycle analysis by flow cytometry was performed 24 h after cardiomyoblasts were treated with anthracyclines. The concentration of each drug that inhibited 50 % proliferation of cultured H9c2(2-1) cells was selected for these experiments. The results of the cell cycle analysis are shown in Fig. 2. The population of untreated control cells contained primarily 2 N content of DNA corresponding to G0/G1 phase (80.1 %). The S and G2/M fractions were 11 and 8.7 %, respectively. Of the H9c2(2-1) cells treated with DOX, 40.6 % showed increased accumulation in G2/M phase of the cell cycle and decreases in G0/G1 and S-phase populations compared with the untreated control. A different effect on the cycling of H9c2(2-1) cells was observed when cells were treated with DOXM and DOXH. These compounds induced increases in the S phase fraction (33.5 and 36.4 % for DOXM and DOXH, respectively). The accumulation of cells in S phase was accompanied by a decline in the G0/G1 population (53.5 and 50.1 % for DOXM and DOXH, respectively) and a slight increase in the percentage of cells in G2/M phase compared with untreated cells. Extremely similar results were obtained for DRB and its formamidine derivatives. DRB-induced cell cycle G2/M arrest resembled the arrest caused by DOX, whereas DRBM and DRBH produced extremely similar patterns of S phase arrest as observed for DOXH and DOXH (data not shown).

### Uptake of anthracyclines by rat cardiomyoblasts

The derivatives were taken up by H9c2(2-1) cells at different rates than those for their parent anthracyclines. Their uptake levels, expressed in nanomoles of compound per 1 × 10^6^ cells, are shown in Table 1. Cellular accumulation of DOX was approximately 30 % higher than its derivative containing a morpholine ring (DOXM). DOXH was transported into the cells more efficiently than DOX and DOXM. The accumulation of DOXH in cardiac myoblasts was almost 2 times higher than that of DOXM and 1.5 times higher than that of the parent doxorubicin. The most “penetrating” compound from the entire group of examined analogs was the daunorubicin derivative with a hexamethyleneimine ring (DRBH); its accumulation in H9c2(2-1) cells was the highest among all tested drugs. After the cells were incubated with this compound for 1 h, the level of accumulation of this compound was more than 1.5 times higher than that of the parent drug (DRB) and at least 5–7 times higher than that of other tested compounds. Our experiments provided conclusive evidence that the hexamethyleneimine moiety enhances the uptake of anthracycline derivatives (DOXH and DRBH) by H9c2(2-1) cells. The lowest uptake values were found for both formamidino derivatives containing a morpholine ring (DOXM and DRBM). The lower cellular uptake values of these compounds translated into decreases in their cytotoxic potency in H9c2(2-1) cells (Table 1).

### Subcellular localization of anthracyclines in rat cardiomyoblasts

**Fig. 3:**
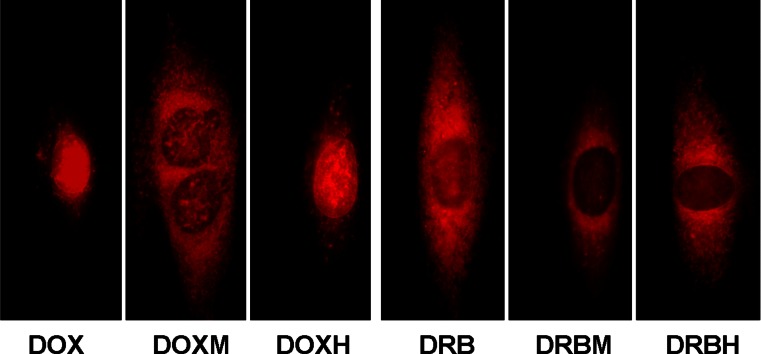
Images of H9c2(2-1) cells incubated with 10 μM anthracycline for 1 h at 37 °C. Cells were observed at 400-fold magnification after excitation with a wide green filter

The fluorescence microscopy images of H9c2(2-1) cells incubated with the compounds are presented in Fig. 3. The anthracyclines accumulated in the nuclei and in the cytoplasm, where they appeared in the form of small granules. As shown in Fig. 3, DOX and DOXH accumulated preferentially in cell nuclei. DOXM exhibited a different preference for subcellular compartments. The red fluorescence corresponding to DOXM was found primarily in the cytoplasm, whereas nuclear chromatin was stained much more weakly. DRB was present in both the nucleus and cytoplasm, whereas its formamidine derivatives DRBM and DRBH were localized primarily in the cytoplasm.

To estimate the percentages of anthracyclines localized in the nucleus and in the cytoplasm of myocardial cells, densitometric quantification was performed. As shown in Table 1, the highest percentage of drug localized in cell nuclei, 80.2 %, was measured for DOX. DOXH exhibited a lower affinity to nuclear chromatin and to the cytoplasm compared to DOX (54.2 % of the DOXH drug was localized in nuclei). All other compounds exhibited the ability to accumulate in the cytoplasm rather than in nuclei. In particular, low affinity to nuclear chromatin was found for the morpholine derivatives DOXM and DRBM as well as for DRBH. All these compounds accumulated primarily in the cytoplasm.

### Correlation between the affinity of anthracyclines to DNA and their localization in the nuclei of rat cardiomyoblasts

**Fig. 4:**
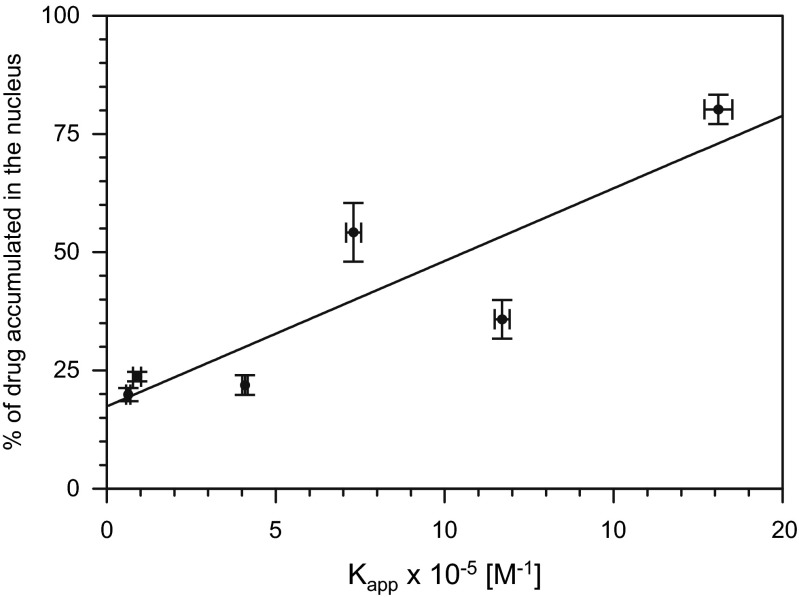
Correlation between the percentage of anthracyclines localized in the nuclei of H9c2(2-1) cells and their DNA binding constants (K_app_). Correlation coefficient r = 0.858, *P* = 0.029. Horizontal and vertical bars represent the standard deviation values

The DNA binding constant (K_app_) values estimated by the ethidium bromide displacement assay are shown in Table 1. The parent compounds DOX and DRB exhibited the highest affinities to double-stranded calf thymus DNA. K_app_ values for their derivatives possessing a hexamethyleneimine ring (DOXH and DRBH) were 2.5–2.8 times lower. The lowest affinities to DNA were found for the morpholine derivatives DOXM and DRBM. When compared to the parent drugs, their K_app_ values were approximately 20 times lower. When we plotted the percentage of anthracyclines localized in the nucleus against their K_app_ values and performed a linear regression analysis, a significant correlation was found (r = 0.858, *P* = 0.029; Fig. 4). Thus, this result indicates that anthracyclines having high affinity to DNA are transported preferably into nuclei.

## Discussion

The delivery and subcellular localization of drugs are important factors that influence their biological activity. Other authors have noted that the cytotoxic activity and clinical efficacy of anthracyclines may strongly correlate with their uptake potency and subcellular distribution [3, 16–19]. In this study, we used cultured rat cardiomyoblasts to compare the uptake, subcellular localization and cytotoxic activity of four anthracycline derivatives and two parent drugs. Our results show that the introduction of a formamidino group with a morpholine or hexamethyleneimine ring at C-3′ of daunosamine reduces the affinity of the tested anthracycline analogs to DNA as well as their cellular uptake and subcellular localization (Table 1). These results confirm the finding of Shaul et al. [3], who found that even small structural changes in the anthracycline molecule affects its cytotoxic activity and subcellular localization. The enhanced uptake of DOXH and DRBH by rat cardiomyoblasts can be explained by their lipophilicity, as the logP values of these derivatives are significantly higher than are those for DOX and DRB (Table 1). This observation is consistent with the findings of other authors who reported the strong impact of lipophilicity on the level of anthracycline accumulation in mammalian cells [16, 18, 19]. We assume that cell morphology is a very important factor that influences drug accumulation. In cardiomyoblasts that contain a large cytoplasm compartment (Fig. 3), the analogs accumulated in the cytoplasm, in contrast to the parent anthracyclines, which were transported preferentially to the nuclei. Our data suggest that cells containing large cytoplasm compartments and small nuclei accumulate hydrophobic anthracyclines preferentially, whereas the hydrophobicities of these drugs are less important for drug accumulation in cells with very large nuclei and small cytoplasmic compartments, e.g., leukemia cells [20].

Cell cycle analysis revealed that the compounds varied in their mode of cytotoxic action (Fig. 2). The growth inhibitory effect of DOX and its formamidine derivatives on cultured cardiomyoblasts appears to be due to blocks at G2/M phase (DOX) and G1/S phase (DOXM and DOXH) of the cell cycle. The same results were obtained for DRB, DRBM and DRBH (data not shown). This result is not surprising because our earlier studies demonstrated that formamidinoanthracyclines are not topoisomerase II inhibitors. These compounds produce different patterns of DNA damage than do their parent compounds, possibly mediated by a free radical mechanism [6, 7], whereas DOX and DRB are known to stabilize the “cleavable complex” of this enzyme with DNA [6, 7, 22].

The structures of the anthracyclines influenced their intracellular distribution. The morpholine as well as hexamethyleneimine substituents reduced the ability of the anthracyclines to bind to nuclear components (Fig. 3 and Table 1). This effect may be explained by the different affinities of the tested anthracyclines to DNA. When the percentages of anthracyclines localized in the nucleus was plotted against their DNA -binding constants, a significant correlation was found (r = 0.858, *P* = 0.029; Fig. 4). Derivatives exhibiting low affinity to DNA may accumulate with higher efficiency in the cytoplasm, as cytoplasmic components such as RNA molecules and proteins may compete with DNA for binding of these drugs.

Affinity to DNA seems to be a primary driving force directing anthracycline molecules into the cell nucleus, and this finding raises the question as to whether nuclear uptake of anthracyclines depends on the DNA content in cells. This possibility exists, as there are cells in different cycle phases and containing different DNA amounts in a population of cardiomyoblasts. Such cell populations should contain cells of different brightness after incubation with fluorescent compounds. However, we did not observe any differences in cell nuclear fluorescence when we viewed several cardiomyoblasts at once in the viewing field at low magnification. The brightness of the nuclei and cytoplasm was dependent on the type of anthracycline derivative, as shown in Fig. 3. This result can likely be explained by the fact that all experiments were performed 24 h after seeding the cells, when the majority of cells were at G0/G1 phase and contained 2 N DNA content (Fig. 2). Nonetheless, this issue is important; therefore, cells that are used to study the intracellular localization of fluorescent drugs should be subjected to cell cycle analysis to ensure that the majority of cells contain similar DNA content.

As mentioned in the Introduction, anthracycline analogs with a modified daunosamine moiety (DOXM, DOXH, DRBM and DRBH) were tested previously in animal models, and all of them exhibited lower cardiotoxicity than did DOX and DRB [4]. Interestingly, in this study, the same group of analogs exhibited decreased cytotoxicity in cultured cardiomyoblasts as well as lowered affinity to nuclear components, presumably primarily to DNA (Table 1 and Fig. 3), when compared to DOX and DRB. The correlation between cytotoxicity in vitro, accumulation of the tested anthracyclines in the nuclei of cardiomyoblasts, and their cardiotoxicity in vivo observed in our previous work [4] leads to the hypothesis that the cytotoxic potencies and the accumulation of anthracyclines in the nuclei of cultured cardiomyoblasts may be good and simple predictors of cardiotoxicity. This is an attractive possibility, as reliable methods that can be used for early prediction of anthracycline cardiotoxicity are needed [21].

The mechanism of anthracycline cardiotoxicity in vivo is very complex, and many cellular metabolic reactions may play a very important role in this process. Therefore, several possible mechanisms of anthracycline cytotoxicity are discussed in the literature [1, 2, 22]. The relatively new concept concerning anticancer drug cardiotoxicity is based on the finding that a small fraction of proliferating cells is present in cardiac tissue and may be a target for antiproliferative agents. [9–11]. The results of our work support this concept, as DOX and DRB, which exhibited the highest accumulation in cell nuclei and were the most potent inhibitors of cardiomyoblast proliferation among the tested compounds, were also found to be the most cardiotoxic in an in vivo animal model.

In conclusion, considering the complexity of the problem, we think that estimating cytotoxic activity and analyzing intracellular distribution of new potential anticancer anthracyclines in cultured cardiomyoblasts may be helpful in their early preclinical evaluation.

## Abbreviations

DOX: doxorubicin
DOXM: doxorubicin containing a formamidino group with a morpholine ring
DOXH: doxorubicin containing a formamidino group with a hexamethyleneimine ring
DRB: daunorubicin
DRBM: daunorubicin containing a formamidino group with a morpholine ring
DRBH: daunorubicin containing a formamidino group with a hexamethyleneimine ring
